# Quality control-driven deep ensemble for accountable automated segmentation of cardiac magnetic resonance LGE and VNE images

**DOI:** 10.3389/fcvm.2023.1213290

**Published:** 2023-09-11

**Authors:** Ricardo A. Gonzales, Daniel H. Ibáñez, Evan Hann, Iulia A. Popescu, Matthew K. Burrage, Yung P. Lee, İbrahim Altun, William S. Weintraub, Raymond Y. Kwong, Christopher M. Kramer, Stefan Neubauer, Hypertrophic Cardiomyopathy Registry (HCMR) Investigators, Oxford Acute Myocardial Infarction (OxAMI) Study, Vanessa M. Ferreira, Qiang Zhang, Stefan K. Piechnik

**Affiliations:** ^1^ Oxford Centre for Clinical Magnetic Resonance Research (OCMR), Division of Cardiovascular Medicine, Radcliffe Department of Medicine, John Radcliffe Hospital, University of Oxford, Oxford, United Kingdom; ^2^ Artificio, Cambridge, MA, United States; ^3^ Faculty of Medicine, University of Queensland, Brisbane, QLD, Australia; ^4^ MedStar Health Research Institute, Georgetown University, Washington, DC, United States; ^5^ Cardiovascular Division, Department of Medicine, Brigham and Women’s Hospital, Harvard Medical School, Boston, MA, United States; ^6^ Department of Medicine, University of Virginia Health System, Charlottesville, VA, United States

**Keywords:** data augmentation, generative adversarial networks, quality control, segmentation, late gadolinium enhancement, virtual native enhancement, cardiovascular magnetic resonance

## Abstract

**Background:**

Late gadolinium enhancement (LGE) cardiovascular magnetic resonance (CMR) imaging is the gold standard for non-invasive myocardial tissue characterisation. However, accurate segmentation of the left ventricular (LV) myocardium remains a challenge due to limited training data and lack of quality control. This study addresses these issues by leveraging generative adversarial networks (GAN)-generated virtual native enhancement (VNE) images to expand the training set and incorporating an automated quality control-driven (QCD) framework to improve segmentation reliability.

**Methods:**

A dataset comprising 4,716 LGE images (from 1,363 patients with hypertrophic cardiomyopathy and myocardial infarction) was used for development. To generate additional clinically validated data, LGE data were augmented with a GAN-based generator to produce VNE images. LV was contoured on these images manually by clinical observers. To create diverse candidate segmentations, the QCD framework involved multiple U-Nets, which were combined using statistical rank filters. The framework predicted the Dice Similarity Coefficient (DSC) for each candidate segmentation, with the highest predicted DSC indicating the most accurate and reliable result. The performance of the QCD ensemble framework was evaluated on both LGE and VNE test datasets (309 LGE/VNE images from 103 patients), assessing segmentation accuracy (DSC) and quality prediction (mean absolute error (MAE) and binary classification accuracy).

**Results:**

The QCD framework effectively and rapidly segmented the LV myocardium (<1 s per image) on both LGE and VNE images, demonstrating robust performance on both test datasets with similar mean DSC (LGE: 0.845±0.075; VNE: 0.845±0.071; p=ns). Incorporating GAN-generated VNE data into the training process consistently led to enhanced performance for both individual models and the overall framework. The quality control mechanism yielded a high performance (MAE=0.043, accuracy=0.951) emphasising the accuracy of the quality control-driven strategy in predicting segmentation quality in clinical settings. Overall, no statistical difference (p=ns) was found when comparing the LGE and VNE test sets across all experiments.

**Conclusions:**

The QCD ensemble framework, leveraging GAN-generated VNE data and an automated quality control mechanism, significantly improved the accuracy and reliability of LGE segmentation, paving the way for enhanced and accountable diagnostic imaging in routine clinical use.

## 1. Introduction

Late gadolinium enhancement (LGE) is a cardiovascular magnetic resonance (CMR) imaging technique that provides crucial information about the extent and location of myocardial damage, allowing clinicians to make accurate diagnoses and treatment decisions (1). It is considered the imaging gold standard for non-invasive myocardial tissue characterisation in a variety of cardiovascular diseases. LGE can identify areas of scar tissue or fibrosis (2), which are often associated with heart disease, such as myocardial infarction (MI) (3) and hypertrophic cardiomyopathy (4). Its quantification provides important information, such as scar-burden, which can predict adverse clinical outcomes like heart failure and sudden death, and may guide risk-modification strategies, such as the use of implantable cardioverter-defibrillator devices (5).

To quantify the extent and location of myocardial pathology in LGE images, the left ventricular (LV) myocardium must be segmented. Manual contouring by experts has been the conventional method, but it is time-consuming and subjective. Recently, there has been growing development in automated segmentation methods to improve efficiency and reduce inter-observer variability. These methods can be broadly categorised as either model-driven (6) or data-driven (7). Model-driven methods use prior knowledge about the structure of the LV myocardium to guide the segmentation process, while data-driven methods use machine learning algorithms to learn from examples in a training dataset, typically yielding superior results than model-driven methods (8). However, despite progress in automated segmentation techniques, clinical translation has been limited by two major challenges. First, data-driven methods require a large amount of high-quality training data (9), which may not always be available, particularly for rare or heterogeneous diseases. Second, even with sufficient training data, unflagged segmentation errors can still occur (10), leading to inaccurate scar quantification, posing a significant concern for clinical decision-making. Thus, there remains a pressing need for a well-validated, automated quality control (QC) mechanism that can detect and flag segmentation errors in a reliable and efficient manner (11).

To overcome the challenge of data scarcity, or limited access, in medical applications, various approaches have been proposed, such as transfer learning, domain adaptation, and data augmentation (12). Transfer learning and domain adaptation aim to leverage knowledge from pre-existing datasets, while data augmentation methods generate new data by applying transformations to existing data. Among these approaches, data augmentation with synthetic data, using Generative Adversarial Networks (GANs), has gained popularity due to its potential to generate large amounts of diverse and realistic data, which can be particularly useful for limited datasets (13, 14). However, the use of synthetic data for medical applications poses a challenge of clinical validation, as the generated data may not accurately reflect the true biological and pathological variations seen in real-world data (15). Therefore, it is crucial to validate the synthetic data before using it for medical purposes.

Automated approaches for flagging inaccuracies in automatic segmentation have gained increasing attention in recent years (11). Post-analysis QC tools have been recently proposed to assess the reliability of segmentation outputs, which are considered the final indicator of a model’s performance. These methods typically act as binary classifiers (16, 17), assigning correct/incorrect labels to a segmentation, or as regressors (18, 19), which attempt to infer well-known validation metrics or uncertainty estimates. While these approaches have been successfully applied to CMR T1 mapping (20) and short-axis cines (21), a QC pipeline for LGE segmentation—an important clinical tool—is still missing.

In this study, we present a novel approach for LGE segmentation that overcomes the challenges of both limited training data and lack of quality control for clinical applications. Our framework leverages the power of GAN-generated data, incorporating virtual native enhancement (VNE) images (22, 23), to further expand the training dataset with clinically-validated data. This emerging contrast-agent-free CMR modality exploits native signals to produce “virtual” LGE images. Additionally, we extend an automated quality control mechanism to flag problematic cases for focused inspection before clinical use. We build upon the quality control-driven (QCD) framework (19, 20), which can predict a confidence metric in absence of ground truth.

## 2. Materials and methods

### 2.1. Imaging data

The development dataset of 4,716 LGE images (1,363 patients) was obtained from the following: (1) the multi-centre Hypertrophic Cardiomyopathy Registry study (24) (HCMR, n=3,286 images from 1,129 patients, 24 centres); (2) the University of Oxford Centre for Clinical Magnetic Resonance Research clinical service (OCMR, n=712 images from 109 patients), and (3) the Oxford Acute Myocardial Infarction study (25) (OxAMI, n=718 images from 125 patients), with institutional review committee and ethics approvals. Altogether, the 4,716 LGE images comprised of 3,286 LGE images from 1,129 patients with hypertrophic cardiomyopathy, and 1,430 LGE images from 234 patients with MI (255 images from 65 patients with chronic MI; 1,175 LGE images from 169 patients with acute MI). CMR scanning was undertaken in Siemens MR scanners (Siemens Healthcare, Germany) with magnetic field strengths of 1.5T (71% of data) and 3T (29% of data). CMR protocols included cine steady-state free precession imaging, native and post-contrast T1 mapping using the ShMOLLI (Shortened Modified Look-Locker Inversion recovery) sequence (26, 27), and LGE imaging acquired at around 10 min after intravenous administration of 0.1 to 0.2 mmol/kg of a gadolinium-based contrast agent, typically with the phase-sensitive inversion recovery sequence (24). Briefly, the manual quality control involved selection of uncorrupted, paired cines, T1 maps and LGE images, which were manually segmented by experienced trained observers (MKB, YPL and IA), in previous studies (22, 23, 28).

### 2.2. Data augmentation using a generative adversarial network

The data were augmented with a conditional generative adversarial network (cGAN) approach to generate VNE images (22, 23) from paired short-axis cine and T1 map. These VNE images exploited native components, including native T1 mapping and pre-contrast cine frames throughout the cardiac cycle. This provided image contrast, alterations in myocardial tissue properties, myocardial structure (such as wall thickness/thickness), motion data of the cardiac wall, and more distinct myocardial borders. The deep learning generator processed these inputs to produce VNE images that closely resembled LGE images in terms of structure and contrast. The clinical utility of VNE lies in its ability to generate “virtual” LGE images without the need for gadolinium, enabling faster, lower-cost, and contrast-free CMR scans.

**Figure 1:**
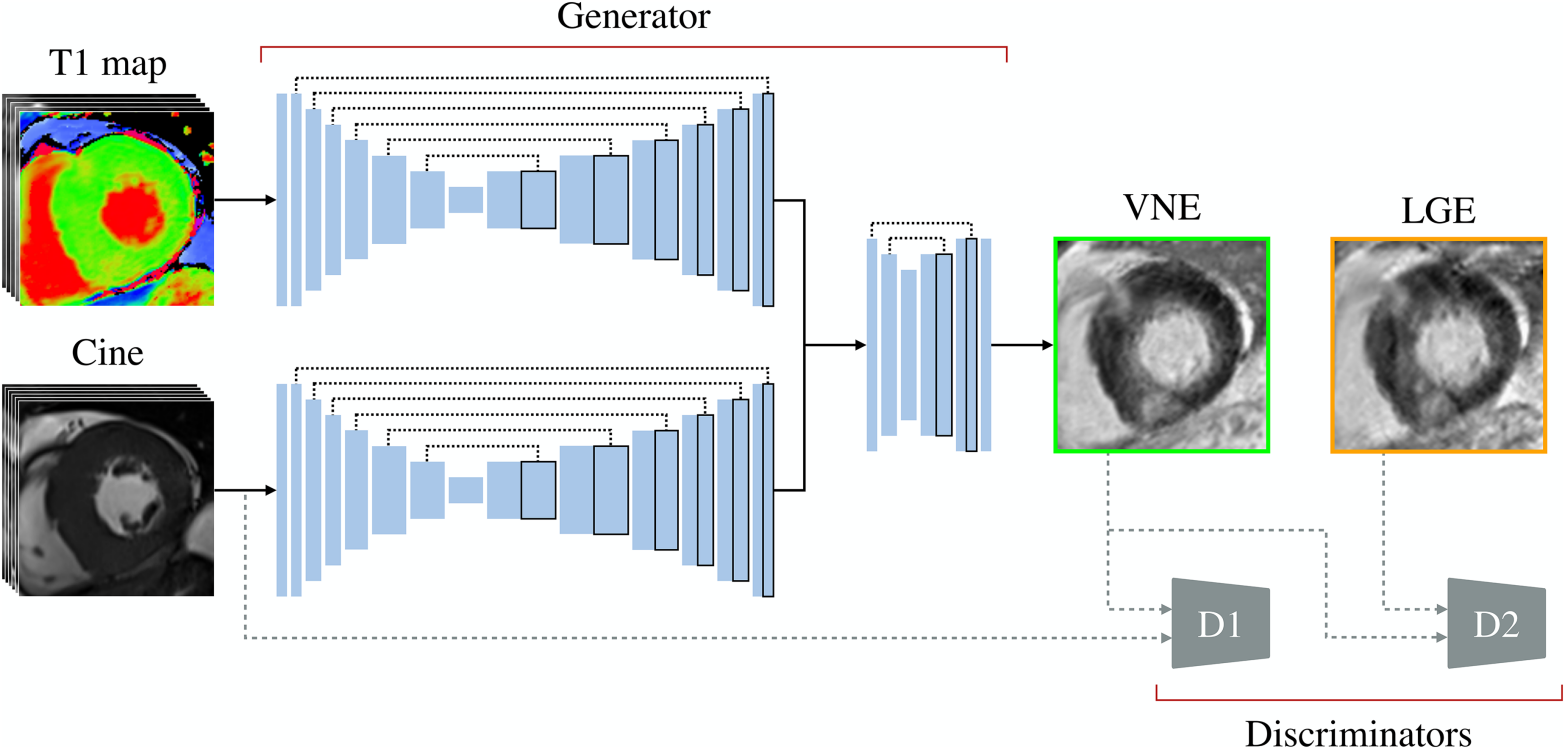
Data augmentation framework. A late gadolinium enhancement (LGE) image is augmented by using its paired short-axis cine and T1 map, producing a virtual native enhancement (VNE) image, using a modified conditional generative adversarial network approach. Parallel deep auto-encoders extract features from native signals, which are fused through a shallow autoencoder to derive a VNE image. The discriminators, D1 and D2, during training, are used to enhance the D1 image “clarity” and the image “realness” with perceptual similarity, respectively.

The VNE generator (Figure 1) consisted of parallel convolutional neural network streams that processed cine frames and motion-corrected T1 maps (29) individually. Each stream utilised a six-level encoder-decoder U-Net structure (30). The encoder computed image features at various scales, with successive convolutional layers for feature extraction and downsampling at each level, offering a multiscale feature representation. The corresponding decoder fused these multiscale features to generate the final feature maps, with symmetrical upsampling layers and convolutions for sequential combination of the multiscale features. These feature maps from the streams were concatenated and fed into an additional two-level encoder-decoder block, which combined information from the different modalities to create the final VNE image in a late fusion manner. Each encoder-decoder block was followed by a tanh activation function.

In the customised cGAN approach (31), the architecture included two discriminators, D1 and D2, modelled after the VGG16 model (32). Discriminator D1, aimed at verifying the “clarity” of larger images, used an expanded architecture with an input layer accommodating the resultant VNE and the input cine stack, which ensured sharper clearer images. This involved a series of convolutional layers, alternating between feature extraction and downsampling, each followed by leaky rectified linear unit activation functions. Discriminator D2, designed to check the “realness” in single-channel images, adopted a similar and more compact structure, processing both the resultant VNE and the paired LGE. The generator’s objective was to create VNE images that had a high perceptual similarity (33) to LGE images and were indiscernible from LGE contrast images. The discriminator’s objective was to differentiate between VNE and LGE images. After training the neural networks in an adversarial manner, we obtained a trained generator capable of translating native CMR signals into LGE-like representations.

**Figure 2:**
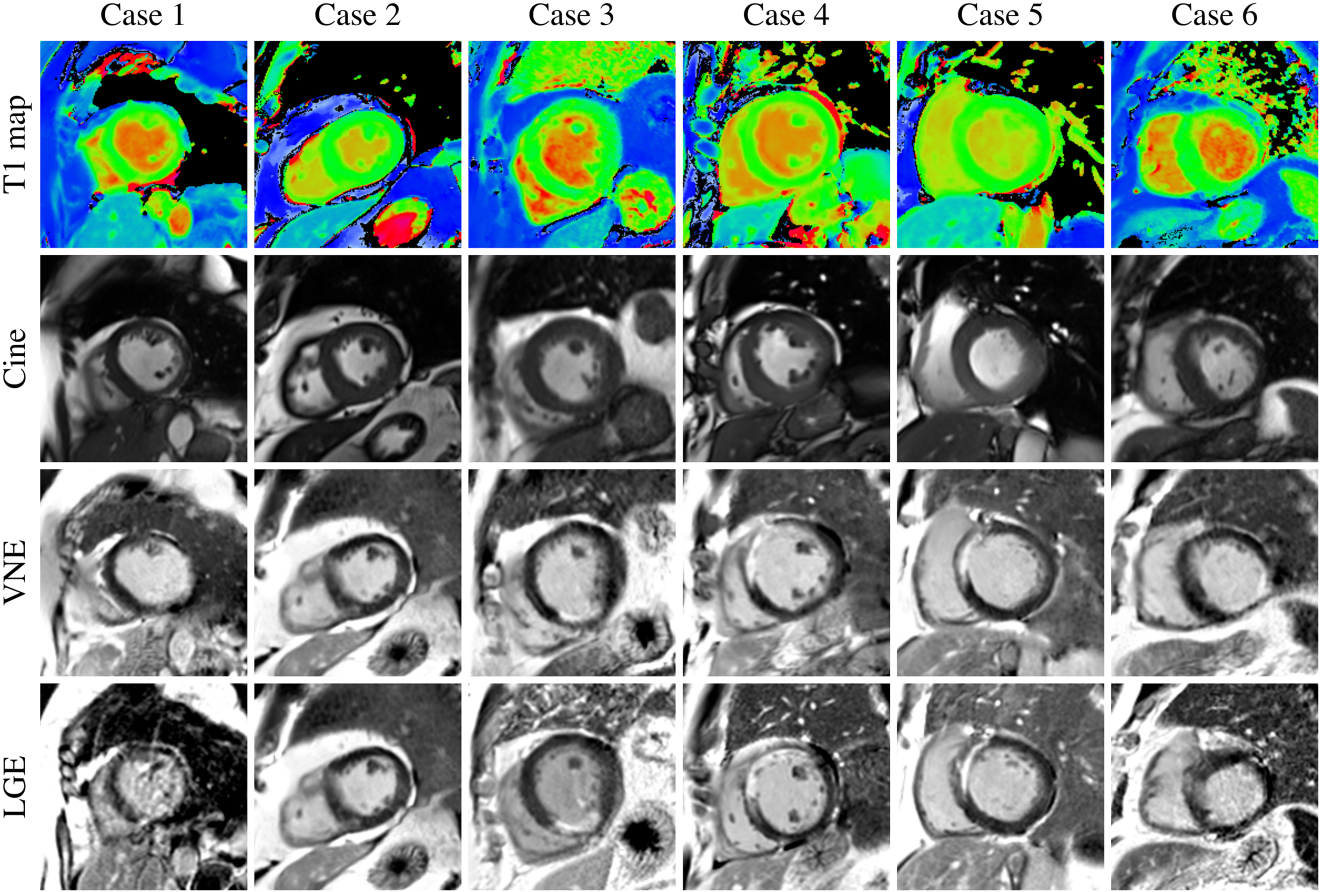
The resultant database includes the late gadolinium enhancement (LGE) data and virtual native enhancement (VNE) data, as ways of data augmentation and additional validation.

With the previously trained VNE generator (22, 23), we expanded the LGE images in the development data by producing corresponding VNE images (Figure 2), in independent datasets. Expansion of the imaging data was successfully carried out for all cases, except for the subset related to acute myocardial infarction, which is awaiting further validation before inclusion. All augmented data were also manually segmented. Through the utilisation of position-matched T1 maps and cine, the derived VNE closely resembled the position-matched LGE; however, in some cases, there were slight differences in slice position between the paired T1/cine and the final LGE, for instance, due to patient movement between the image acquisitions (Figure 2, cases 5 and 6). Serendipitously, this introduced increased diversity and realism into the training data, thereby enhancing the robustness of the model.

### 2.3. Quality control-driven ensemble framework

**Figure 3:**
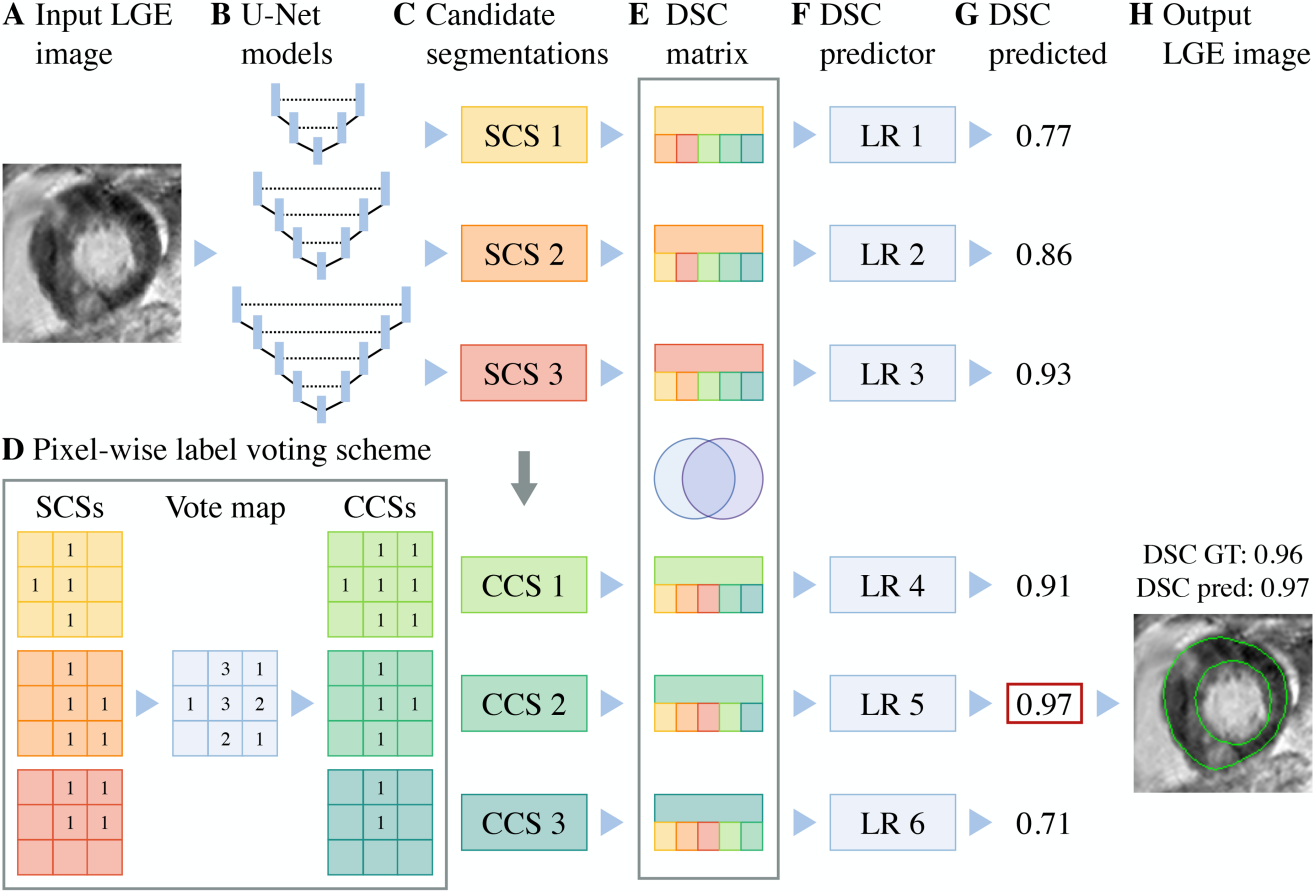
Illustrative quality control-driven ensemble framework depicted with 3 (out of 6) U-Nets. (**A**) A late gadolinium enhancement (LGE) image is processed by (**B**) an ensemble of independent U-Net segmentation models to produce (**C**) single candidate segmentations (SCSs). (**D**) The SCSs are then combined via a pixel-wise label voting scheme to derive combined candidate segmentations (CCSs). (**E**) An association matrix of Dice Similarity Coefficients (DSC) is generated upon the agreement between SCSs and CCSs. The inter-candidate DSCs are supplied to the (**F**) linear regressors (LR), and (**G**) each model outputs the predicted the DSC, in absence of ground truth (GT); finally, (**H**) the model with the highest predicted DSC and its corresponding automated segmentation output are selected on-the-fly.

A quality control-driven ensemble framework (19) (Figure 3) was developed to enhance the accuracy and reliability of the segmentation process by leveraging the strengths of multiple convolutional neural networks. This framework utilised various U-Nets (30) with different depths to create a diverse set of candidate segmentations. These segmentations were then combined using statistical rank filters in a pixel-wise fashion (34), further expanding the pool of segmentation candidates and improving robustness.

In the ensemble framework, six U-Nets (30), with depths ranging from 1 to 6 levels, were employed. Each U-Net consisted of an encoder and a decoder. The encoder had convolutional layers followed by dropout layers (35) for regularisation, with increasing dropout rate with each layer to mitigate overfitting. Post-convolution and dropout, a max pooling operation was applied. The decoder mirrored the encoder but used transposed convolutional layers for upscaling. It also utilised skip connections, coupling outputs from the decoder with corresponding encoder layers. The final layer underwent additional convolutions and a softmax activation to produce the final segmentation. The overall process, which was repeated for each depth, allowed the generation of diverse candidate segmentations, contributing to the ensemble’s performance.

The automatic quality scoring mechanism at the core of the framework predicted the Dice Similarity Coefficient (DSC) for each candidate segmentation by exploiting their differences. It calculated the pairwise agreement, or inter-segmentation DSC matrix, between segmentations, capturing the overlap and divergence between different candidates. These DSC matrices were then fed into separate linear regression models for each candidate, with the target being the DSC between the candidate and the ground truth.

For each input image, the framework assigned a predicted DSC, with respect to the ground truth segmentation, to every candidate segmentation, both single and combined. The final segmentation was selected by identifying the candidate with the highest predicted DSC, indicating the most accurate and reliable result. This selection process was performed automatically by the framework, without any manual intervention. This approach effectively emulated a multidisciplinary clinical team, where the consistency among multiple expert opinions served as a marker for the best approach in managing complex cases. By incorporating this quality control-driven strategy, the ensemble framework aimed to improve overall segmentation performance and provide confidence metrics, particularly useful in clinical settings.

### 2.4. Implementation

The data were augmented with the VNE technology using available co-located short-axis cines and ShMOLLI T1 maps, resulting in 3,541 VNE images. The development dataset was randomly partitioned into: (1) 85% for the training dataset (4,092 LGE images and 2,917 VNE images from 1,158 patients); (2) 7.5% for validation (309 LGE/VNE images from 102 patients); (3) 7.5% for the test dataset (309 LGE/VNE images from 103 patients), per recommended guidelines (36). Image pixel values were scaled from 0 to 1 and zero-padded to 256×256. For the segmentation models, the Adam method (37) was used for optimising the categorical cross-entropy loss, with a learning rate of $5\times 10^{-5}$ for 200 epochs; an automated early stop was used to avoid overfitting, using the validation set. For the quality prediction models, a linear regressor for each candidate segmentation was fit with the inter-agreement between its corresponding candidate segmentation and the rest. These regressors were trained on the validation set to avoid autocorrelation with the training set. The models were trained and tested on TensorFlow (38) with an NVIDIA GeForce RTX 3090 GPU, taking approximately 11.5 h.

### 2.5. Evaluation

The performance of the QCD ensemble framework was evaluated for myocardial contours on both LGE and VNE test datasets, and across the main pathologies. The segmentation accuracy was assessed by DSC, comparing the agreement between the optimally selected mask and the ground-truth mask. The predicted segmentation accuracy was assessed in terms of the mean absolute error (MAE) and binary classification accuracy, with a DSC threshold at 0.7 (39). The former was used to measure the difference between the predicted DSC and the observed ground-truth DSC derived from the manual segmentation. The latter assessed whether the segmentations were classified into good (≥0.7) or poor quality (<0.7) to demonstrate the practical usage of the DSC prediction. In the evaluation, false positives occurred when the predicted DSC was ≥0.7 but the real accuracy was <0.7, while false negatives arose when the predicted DSC was <0.7 despite the real accuracy being ≥0.7. The threshold of 0.7 ensured a balance between sensitivity and specificity in the classification of good and bad quality segmentations. As evidenced in our prior works on aortic (19) and myocardial segmentation (20, 40), this threshold provided a standard measure of segmentation quality across different studies. In this context, a DSC of 0.7 implied that 70% of the segmentation correctly overlapped with the ground truth, which was considered to be an acceptable level of accuracy for our applications. A Wilcoxon signed-rank test was conducted using Python to determine if there was a statistically significant difference between the segmentation results obtained on LGE data and VNE data, paired when possible, and within pathology groups. P<0.05 was considered significant. This analysis helped to ascertain the robustness of the model when segmenting both types of images and the potential benefits of incorporating VNE data into the training process.

#### 2.5.1. Comparative analysis

A comparative study was conducted to investigate the pipeline’s main components performance and the impact of incorporating VNE data. Firstly, the performance of the deepest, top-performing employed U-Net (depth of 6 levels) and the QCD segmentation framework were assessed to highlight the benefit of a higher segmentation accuracy with a quality predictive capacity. Secondly, transversely, each experiment involved training with LGE data, VNE data, and both combined, to thoroughly evaluate the data augmentation capability of the GAN-generated VNE data. Thirdly, each experiment was also tested on LGE data, VNE data, and both combined, to exhibit the robustness of the proposed method. The segmentation accuracy and the quality prediction accuracy were assessed in all experiments, to compare the differences. Lastly, the extensive data augmentation techniques, proposed in the nnU-Net framework (41) were also implemented, to assess the added benefit of VNE data in the pipeline.

## 3. Results

### 3.1. Segmentation and prediction accuracy

**Figure 4:**
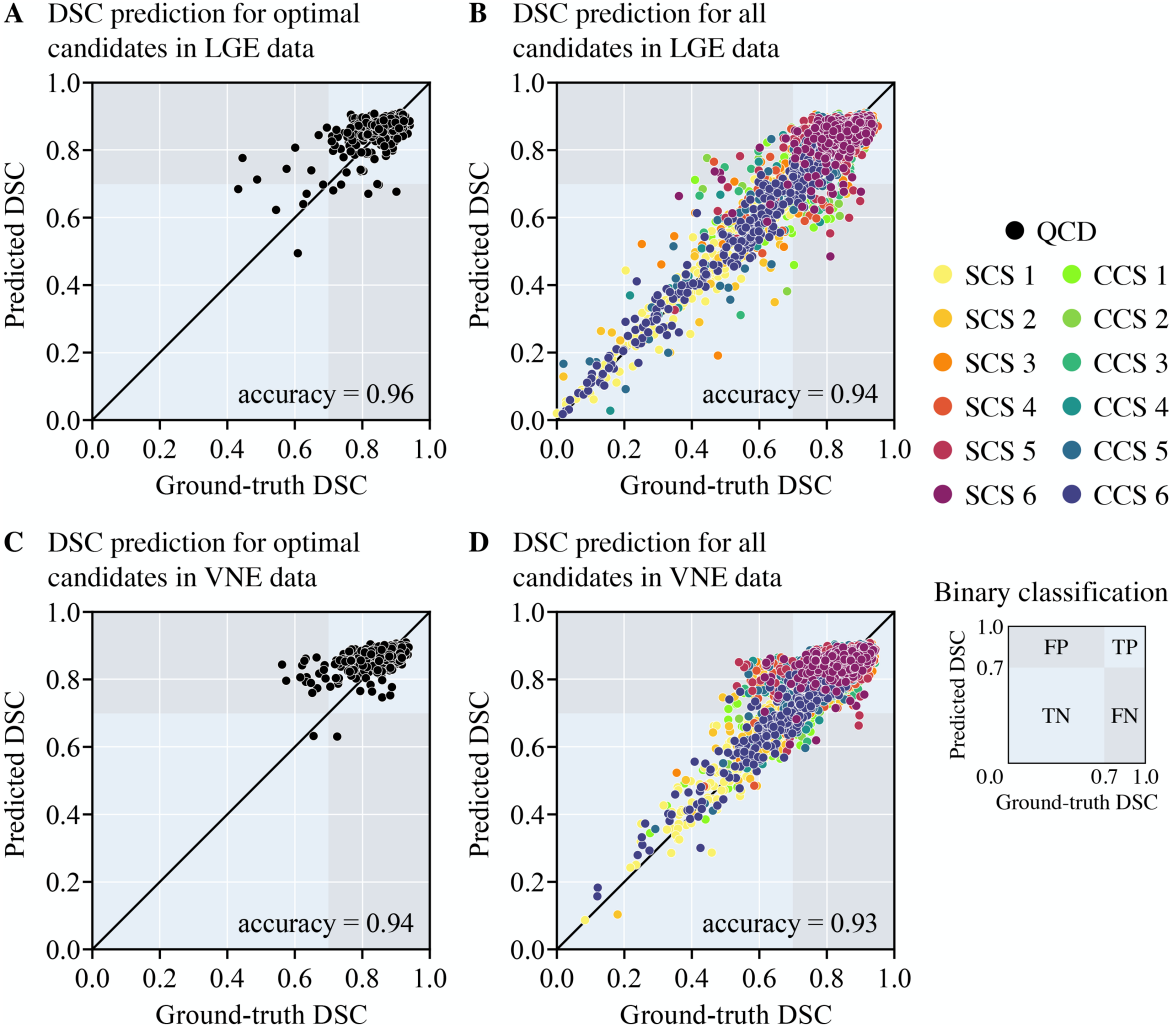
Scatter plots of the observed ground-truth Dice Similarity Coefficient (DSC) (x-axis) versus the predicted DSC (y-axis) for myocardial contours in (**A**,**B**) late gadolinium enhancement (LGE) and (**C**,**D**) virtual native enhancement (VNE) images for the optimal candidates (**A**,**C**) and for all single (SCS) and combined segmentation (CCS) models (**B**,**D**). The shown overall binary classification accuracy is measured as the proportion of true results (true positive (TP) or true negative (TN)—light blue background), in a population of both true and negative results (false positive (FP) or false negative (FN)—grey background), with a binary threshold of DSC ≥0.7.

**Figure 5:**
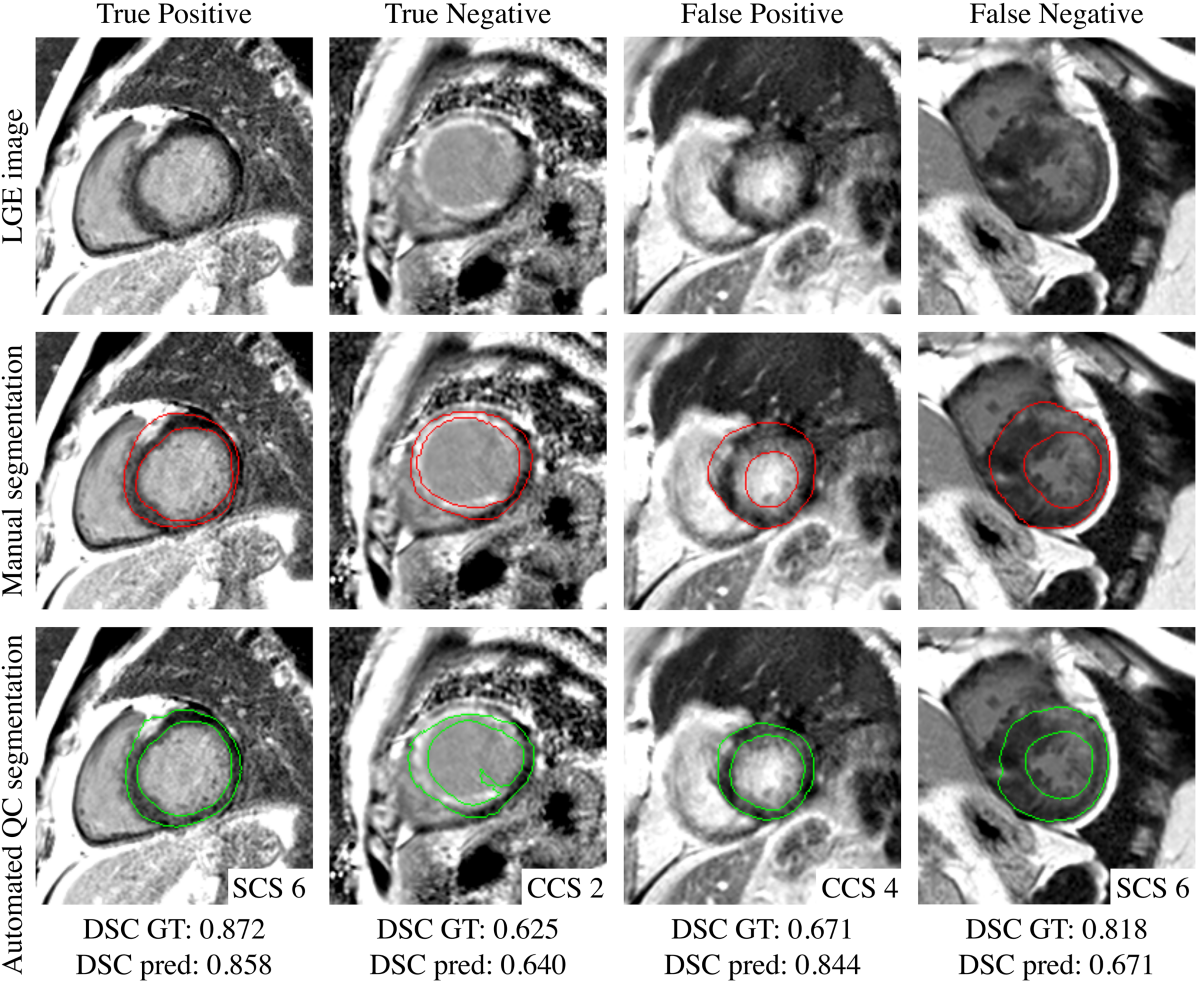
Examples of true positive (93.9%), true negative (1.9%), false positive (2.3%) and false negative (1.9%) for predicted quality-controlled (QC) segmentations in late gadolinium enhanced (LGE) images. The left ventricular myocardium is manually segmented in red and automatically segmented in green, from different single (SCS) and combined candidate segmentation (CCS) models. The corresponding observed ground-truth (GT) Dice Similarity Coefficient (DSC) and predicted DSC are provided at the bottom.

**Figure 6:**
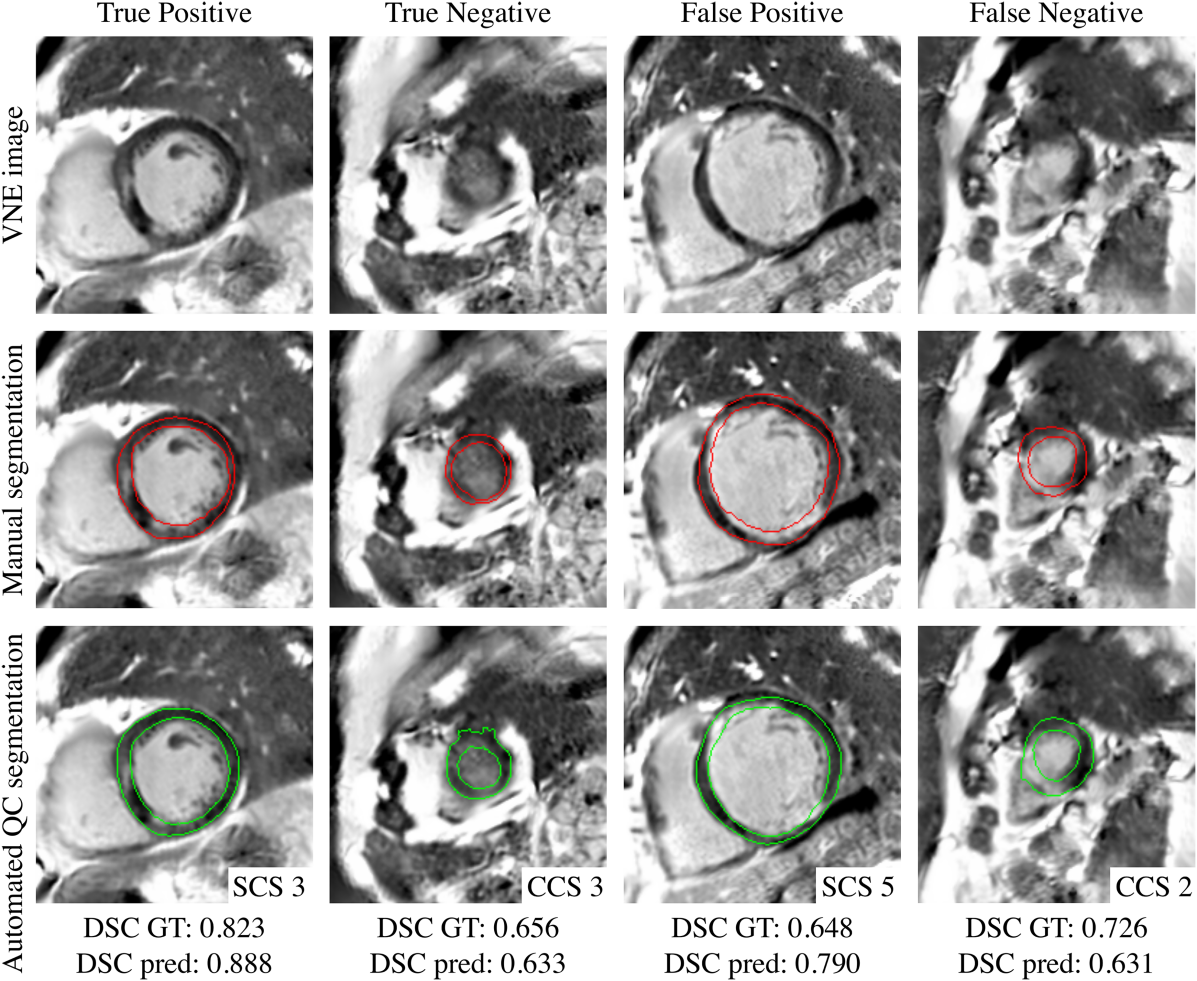
Examples of true positive (94.2%), true negative (0.3%), false positive (5.2%) and false negative (0.3%) for predicted quality-controlled (QC) segmentations in virtual native enhancement (VNE) images. The left ventricular myocardium is manually segmented in red and automatically segmented in green, from different single (SCS) and combined candidate segmentation (CCS) models. The corresponding observed ground-truth (GT) Dice Similarity Coefficient (DSC) and predicted DSC are provided at the bottom.

The scatter plots (Figure 4) reflect the parity between the ground-truth DSC and the predicted DSC for the resultant framework output and for every candidate model output in the test set, being able to accurately predict from underperforming models to highperforming models. The QCD framework successfully and rapidly segmented the LV myocardium on LGE and VNE images. The QCD framework demonstrated robust segmentation performance on both LGE and VNE test datasets, with similar mean DSC (LGE: 0.845±0.075; VNE: 0.845±0.071; p=ns). The QCD framework also exhibited robust segmentation performance across the main pathologies (hypertrophic cardiomyopathy: 0.845±0.069; MI: 0.844±0.085; p=ns). The mean absolute error (MAE) for the predicted DSC was low at 0.043±0.043, demonstrating the accuracy of the quality control-driven strategy in predicting the segmentation quality. Moreover, using the DSC threshold of 0.7, the binary classification accuracy was high at 0.951, further emphasising the practical usefulness of the proposed ensemble framework in clinical settings. Figures 5 and 6 show representative test cases of the QCD framework on LGE and VNE images, respectively, for true positive, true negative, false positive and false negative cases.

### 3.2. Comparative analysis

The comparative analysis (Table 1) highlights the contribution of the GAN-generated VNE as data augmentation and the QCD segmentation framework as an automated quality control mechanism. Firstly, the individual performance of the deepest U-Net was on par with the ensemble performance of the QCD segmentation framework, which was also able to estimate the quality of the resultant segmentation. When analysing the results using all datasets for training and testing, the deepest U-Net achieved a segmentation accuracy of 0.845±0.070, similar to the segmentation accuracy of the QCD segmentation framework. Secondly, the performance of the deepest U-Net, trained on only LGE data, completely generalised to the VNE test set, with a DSC of 0.836±0.082 and 0.838±0.075 for the LGE and VNE test sets, respectively. Similarly, the deepest U-Net, trained on only VNE data, with 30% less data, also generalised to the LGE test set, with a DSC of 0.791±0.119 and 0.824±0.084 for the LGE and VNE test sets, respectively. These findings, similar to the ones with the ensemble framework, effectively support the resemblance of VNE to LGE images, and validates the data augmentation approach for virtually yielding the same performance as its counterpart.

**Table 1:** Comparative analysis of individual models (U-Net with depth of 6 levels) and quality control-driven (QCD) segmentation framework.

Models and traning data		Test data								
		LGE (n=309)			VNE (n=309)			LGE + VNE (n=618)		
Models	Training set	DSC	MAE	Acc.	DSC	MAE	Acc.	DSC	MAE	Acc.
U-Net	LGE	0.836	—	—	0.838	—	—	0.837	—	—
U-Net	VNE	0.791	—	—	0.824	—	—	0.807	—	—
U-Net	LGE + VNE	0.844	—	—	**0.846**	—	—	0.845	—	—
QCD	LGE	0.835	**0.042**	**0.971**	0.838	0.046	0.922	0.837	0.044	0.947
QCD	VNE	0.799	0.057	0.922	0.833	**0.041**	**0.958**	0.816	0.049	0.940
**QCD**	**LGE + VNE**	**0.845**	0.042	0.958	0.845	0.043	0.945	**0.845**	**0.043**	**0.951**

The models were trained on late gadolinium enhancement (LGE; n=4,092) and/or virtual native enhancement (VNE; n=2,917) data and tested on LGE (n=309) and/or VNE (n=309) data, evaluated by their segmentation performance with the mean Dice Similarity Coefficient (DSC) and the quality predictive capacity with the mean absolute error (MAE) and the binary classification accuracy (Acc.). The best results are highlighted in bold.

Thirdly, including the GAN-generated data consistently improved the performance in both experiments of the individual model and the QCD segmentation framework, in every experiment. For instance, the QCD segmentation framework trained on only LGE data yielded a mean DSC of 0.835±0.082 and 0.838±0.080 for the LGE and VNE test sets, respectively; whereas the QCD segmentation framework trained on both LGE and VNE data yielded a higher segmentation performance of 0.845±0.075 and 0.845±0.071, indicating the benefits of including VNE data in the training process for the segmentation accuracy. The quality predictive capacity also improved when trained and tested in both datasets. Lastly, the individual segmentation performance of the model trained with only LGE data including extensive data augmentation (41) (0.846±0.072) was also surpassed by the same framework when the VNE data were added (0.851±0.068). Overall, no significant differences were observed when comparing LGE and VNE test sets between the experiments.

## 4. Discussion

In this study, we demonstrated that the proposed framework, leveraging GAN-generated VNE data and incorporating an automated quality control mechanism, significantly improved the accuracy and reliability of LGE segmentation. The comparative analysis demonstrated the benefits of using VNE data, with generally better image quality and more consistency (22, 23), in the training process and the effectiveness of the ensemble framework in enhancing segmentation performance. Moreover, the proposed framework revealed robustness in both LGE and VNE data. This represents an accountable pipeline for automated segmentation in the gold-standard and emerging contrast-agent-free modalities, paving the way for faster and reliable diagnosis of myocardial damage.

Data scarcity or lack of access remains a persistent challenge in developing robust and reliable deep learning models for medical image segmentation, particularly in the context of LGE segmentation. This limitation stems from the high costs and ethical considerations associated with acquiring, labelling, and sharing large-scale annotated datasets, which often result in insufficient representation of diverse and rare pathological cases (9). As a consequence, models may underperform or fail to generalise well to unseen cases (10), hampering their clinical utility. The application of generating VNE images (22, 23) assured a data augmentation technique with more reliable image contrast. This significantly improves over prior methods of using synthetic LGE images (42, 43), which had not been clinically-validated and were not designed for displaying LGE lesion signals. Addressing data scarcity through the generation of VNE images, as demonstrated in this study, can alleviate this issue by augmenting the already-available LGE data, or potentially substituting the data, leading to a more robust and reliable model that can better handle the complexity of clinical cases and ultimately improve patient care.

The presented work focused on the integration of an automated quality assurance framework, which was developed with a traditional encoder-decoder U-Net architecture (30) with different depths. The quality control-driven strategy provided reliable quality predictions, crucial for clinical decision-making. The adapted regression-based quality prediction scheme enables further exploitation of the diversity of different candidates, with a deterministic approach of assessing the agreement between candidates, proven to be more effective than the emerging Monte Carlo-based quality assurance scheme (40). Newer network architectures and advanced pre-processing schemes could be incorporated to increase such diversity, ranging from existing LGE segmentation approaches (44) to spatial transformation-based pre-processing (45, 46). Future work will cover dataset extension, different candidate models, and scar burden evaluation.

The clinical implications of the proposed QCD ensemble framework are substantial, as it introduces an automated quality control mechanism for the first time in automated LGE segmentation, improving both accuracy and reliability. Moreover, the quality control-driven strategy allows for the identification and refinement of suboptimal segmentations, ensuring the system’s efficiency and trustworthiness, paving the way for increased adoption in clinical workflows. This enhancement streamlines the diagnostic process, reduces contouring variability, and bolsters clinician confidence in automated segmentation results, potentially leading to easier scar burden quantification on a routine basis, better-informed treatment decisions and improved patient outcomes.

This work has some limitations. The main goal has been the fundamental task of the myocardial delineation, thus avoiding engaging the generalisation into the known difficulties of scar tissue quantification (47). In particular the diffuse, less structured and scattered lesions require lower segmentation thresholds, and the targets are subject to many methodological choices and biases in ground truth data (48, 49). While our model was evaluated on an extensive international database, enabling potential generalisation to various conditions requiring LGEs, the findings have been primarily concentrated on patients with hypertrophic cardiomyopathy and MI, suggesting the need for future validation across a wider spectrum of pathologies. Lastly, our selection of the DSC threshold of 0.7 for discerning between acceptable and unacceptable segmentations, despite being effective in our prior work (19, 20, 40), might not address all the geometric properties of cardiac structures (10). We expect that proposed methods would generalise well to any single particular threshold or application, yet further research is needed to explore the available choices for the clinical routine use beyond the scope of this work.

## 5. Conclusion

In conclusion, our study presents a novel approach for automated LGE segmentation that overcomes the challenges of limited training data and lack of quality control, for clinical applications. By leveraging the power of GAN-generated VNE images and incorporating an automated quality control mechanism, we demonstrate the potential for improved automated segmentation performance and reliability. This framework could be seamlessly integrated into clinical studies, providing an efficient, quality-controlled and reliable tool for clinicians in diagnosing and managing patients with myocardial damage.

## Data Availability

The data analysed in this study is subject to the following licenses/restrictions: HCMR, OCMR and OxAMI studies can be approached independently for data sharing upon reasonable request. De-identified clinical imaging data can be shared with interested parties, subject to ethical approval, consent, data-protection governance, data sharing agreements and funding available. Requests to access these datasets should be directed to HCMR, https://hcmregistry.org; OCMR, https://www.rdm.ox.ac.uk; OxAMI, https://oxami.org.uk.
