# Supplementary material for: Quality control-driven deep ensemble for accountable automated segmentation of cardiac magnetic resonance LGE and VNE images
Source: Front Cardiovasc Med. 2023 Sep 11;10:1213290. doi: 10.3389/fcvm.2023.1213290 (PMC10518404; doi:10.3389/fcvm.2023.1213290)
Supplement: Supplementary file 1 [file Datasheet1.pdf]

Supplementary Material

1 HYPERTROPHIC CARDIOMYOPATHY REGISTRY (HCMR) INVESTIGATORS

(In alphabetical order)

Theodore Abraham, Hypertrophic Cardiomyopathy Center of Excellence, Johns Hopkins University, Baltimore, United States of America;

Lisa Anderson, St George's University Hospitals NHS Trust, London, United Kingdom;

Florian Andre, Department of Medicine, Heidelberg University, Heidelberg, Germany;

Evan Appelbaum, Departments of Medicine, Cardiovascular Division & Radiology, Beth Israel Deaconess Medical Center, Harvard Medical School, Boston, United States of America;

Camillo Autore, Division of Cardiology, Department of Clinical & Molecular Medicine, St. Andrea Hospital, Sapienza University, Rome, Italy;

Colin Berry, British Heart Foundation Glasgow Cardiovascular Research Center, Institute of Cardiovascular & Medical Sciences, University of Glasgow, United Kingdom;

Elena Biagini, Cardio-Thoraco-Vascular Department, University Hospital of Bologna, Policlinico S. Orsola-Malpighi, Bologna, Italy;

William Bradlow, Department of Cardiology, New Queen Elizabeth Hospital Birmingham, United Kingdom;

Chiara Bucciarelli-Ducci, Bristol Heart Institute, Bristol National Institute of Health Research (NIHR) Biomedical Research Center, University Hospitals Bristol NHS Trust & University of Bristol, United Kingdom;

Amedeo Chiribiri, Cardiovascular Division, Kings College London British Heart Foundation Center of Excellence, The Rayne Institute, St. Thomas Hospital Campus, London, United Kingdom;

Lubna Choudhury, Division of Cardiology, Department of Medicine, Bluhm Cardiovascular Institute, Northwestern University Feinberg School of Medicine, Chicago, United States of America;

Andrew Crean, Division of Cardiology, Peter Munk Cardiac Center, University Health Network, University of Toronto, Ontario, Canada;

Dana Dawson, Aberdeen Cardiovascular & Diabetes Center, University of Aberdeen, United Kingdom;

Milind Y. Desai, Department of Cardiovascular Medicine, Center for Radiation Heart Disease, Heart & Vascular Institute, Cleveland Clinic, Cleveland, United States of America;

Patrice Desvigne-Nickens, National Heart, Lung, and Blood Institute, Bethesda, United States of America;

John DiMarco, University of Virginia Health System, Charlottesville, United States of America;

Eleanor Elstein, Division of Cardiology, Department of Medicine, Royal Victoria Hospital, McGill University Health Center, Montreal, Quebec, Canada;

Andrew Flett, Department of Cardiology, University Hospital Southampton NHS Foundation Trust, Southampton, United Kingdom;

Nancy Geller PhD, National Heart, Lung, and Blood Institute; Stephen Heitner, Oregon Health & Sciences University (OHSU), Division of Cardiovascular Medicine, Knight Cardiovascular Institute, Portland, United States of America;

Adam Helms, Department of Internal Medicine, University of Michigan, Ann Arbor, United States of America;

Carolyn Ho, Cardiovascular Division, Brigham and Womens Hospital, Boston, United States of America;

Daniel L. Jacoby, Section of Cardiovascular Medicine, Department of Internal Medicine, Yale School of Medicine, New Haven, United States of America;

Han Kim, Duke Cardiovascular Magnetic Resonance Center & Division of Cardiology, Duke University Medical Center, Durham, United States of America;

Bette Kim, Mount Sinai West, Icahn School of Medicine at Mount Sinai, New York City, United States of America;

Dong-Yun Kim PhD, National Heart, Lung, and Blood Institute; Eric Larose, Quebec Heart & Lung Institute, Laval University, Quebec, Canada;

Masliza Mahmod, Division of Cardiovascular Medicine, Radcliffe Department of Medicine, University of Oxford, United Kingdom; Heiko Mahrholdt, Department of Cardiology, Robert-Bosch-Krankenhaus, Stuttgart, Germany;

Martin Maron, Hypertrophic Cardiomyopathy Center & Research Institute, Tufts Medical Center, Boston, United States of America;

Gerry McCann, Department of Cardiovascular Sciences, University of Leicester, United Kingdom; Michelle Michaels, Erasmus University, Rotterdam, the Netherlands;

Saidi Mohiddin, Barts Heart Center, The Cardiovascular Magnetic Resonance Imaging Unit, St Bartholomew's Hospital, London, United Kingdom;

Sherif Nagueh, Methodist DeBakey Heart & Vascular Center, Houston, United States of America;

David Newby, Center for Cardiovascular Science, University of Edinburgh, United Kingdom;

Iacopo Olivotto, Cardiomyopathy Unit & Genetic Unit, Careggi University Hospital, Florence, Italy;

Anjali Owens, Center for Inherited Cardiovascular Disease, Division of Cardiovascular Medicine, Perelman School of Medicine, University of Pennsylvania, Philadelphia, United States of America;

F. Pierre-Mongeon, Montréal Heart Institute, Canada; Sanjay Prasad, National Heart & Lung Institute, Imperial College London & Royal Brompton Hospital, London, United Kingdom;

Ornella Rimoldi, Vita Salute University & San Raffaele Hospital, Milan, Italy;

Jeanette Schulz-Menger, Charité, Medical Faculty of the Humboldt University, Experimental & Clinical Research Center and Helios Clinics, Cardiology, Berlin, Germany;

Mark Sherrid, Hypertrophic Cardiomyopathy Program, Leon Charney Division of Cardiology, Department of Medicine, New York University School of Medicine, New York, United States of America;

Sven Plein, Department of Biomedical Imaging Science, Leeds Institute of Cardiovascular and Metabolic Medicine, University of Leeds, United Kingdom;

Albert van Rossum, Department of Cardiology, Amsterdam UMC, HZ Amsterdam, the Netherlands;

Jonathan Weinsaft, Departments of Medicine & Radiology, Weill Cornell Medical College, New York, United States of America;

James White, Calgary Foothills Medical Center, University of Calgary, Alberta, Canada;

Eric Williamson, Department of Radiology, Mayo Clinic, Rochester, United States of America.

2 OXFORD ACUTE MYOCARDIAL INFARCTION (OXAMI) STUDY INVESTIGATORS

(In alphabetical order)

Adrian P. Banning, Acute Vascular Imaging Centre (AVIC), University of Oxford, Oxford University Hospitals NHS Trust, John Radcliffe Hospital, Oxford, United Kingdom;

Keith Channon, Acute Vascular Imaging Centre (AVIC), University of Oxford, Oxford University Hospitals NHS Trust, John Radcliffe Hospital, Oxford, United Kingdom;

Robin P. Choudhury, Acute Vascular Imaging Centre (AVIC), University of Oxford, Oxford University Hospitals NHS Trust, John Radcliffe Hospital, Oxford, United Kingdom;

Giovanni L. De Maria, Acute Vascular Imaging Centre (AVIC), University of Oxford, Oxford University Hospitals NHS Trust, John Radcliffe Hospital, Oxford, United Kingdom;

Raj Kharbanda, Oxford University Hospitals NHS Trust, John Radcliffe Hospital, Oxford, United Kingdom;

Jeremy Langrish, Oxford University Hospitals NHS Trust, John Radcliffe Hospital, Oxford, United Kingdom;

Andrew Lucking, Oxford University Hospitals NHS Trust, John Radcliffe Hospital, Oxford, United Kingdom.
